# Development of the Systems Thinking for Health Actions framework: a literature review and a case study

**DOI:** 10.1136/bmjgh-2022-010191

**Published:** 2023-03-17

**Authors:** Jenna Thelen, Carmen Sant Fruchtman, Muhammad Bilal, Kebabonye Gabaake, Shahid Iqbal, Tshiamo Keakabetse, Aku Kwamie, Ellen Mokalake, Lucia Mungapeyi Mupara, Onalenna Seitio-Kgokgwe, Shamsa Zafar, Daniel Cobos Muñoz

**Affiliations:** 1 Epidemiology and Public Health, Swiss Tropical and Public Health Institute, Allschwil, Switzerland; 2 University of Basel, Basel, Switzerland; 3 Public Health, Child Advocacy International, Islamabad, Pakistan; 4 Public Health, Institute of Development Management, Gaborone, Botswana; 5 Alliance for Health Policy and Systems Research, World Health Organization, Geneve, Switzerland; 6 Monitoring Evaluation and Quality Assurance, Ministry of Health Botswana, Gaborone, Botswana; 7 Department of Obstetrics and Gynecology, Fazaia Medical College, Islamabad, Pakistan

**Keywords:** COVID-19, Health systems, Qualitative study

## Abstract

**Background:**

Systems thinking is an approach that views systems with a holistic lens, focusing on how components of systems are interconnected. Specifically, the application of systems thinking has proven to be beneficial when applied to health systems. Although there is plenty of theory surrounding systems thinking, there is a gap between the theoretical use of systems thinking and its actual application to tackle health challenges. This study aimed to create a framework to expose systems thinking characteristics in the design and implementation of actions to improve health.

**Methods:**

A systematised literature review was conducted and a Taxonomy of Systems Thinking Objectives was adapted to develop the new ‘Systems Thinking for Health Actions’ (STHA) framework. The applicability of the framework was tested using the COVID-19 response in Pakistan as a case study.

**Results:**

The framework identifies six key characteristics of systems thinking: (1) recognising and understanding interconnections and system structure, (2) identifying and understanding feedback, (3) identifying leverage points, (4) understanding dynamic behaviour, (5) using mental models to suggest possible solutions to a problem and (6) creating simulation models to test policies. The STHA framework proved beneficial in identifying systems thinking characteristics in the COVID-19 national health response in Pakistan.

**Conclusion:**

The proposed framework can provide support for those aiming to applying systems thinking while developing and implementing health actions. We also envision this framework as a retrospective tool that can help assess if systems thinking was applied in health actions.

**WHAT IS ALREADY KNOWN ON THIS TOPIC**

- Systems thinking is a discipline that has proven useful in addressing problems in health systems strengthening.

**WHAT THIS STUDY ADDS**

- The Systems Thinking for Health Actions framework simplifies the application of systems thinking in health actions by providing an operational guide.

**HOW THIS STUDY MIGHT AFFECT RESEARCH, PRACTICE OR POLICY**

- This study intends to increase the use of systems thinking implemented in health actions and uncover systems thinking methods that are already being applied in health actions.

## Introduction

Complex adaptive systems (CAS) are systems that contain a myriad of intricately interconnected components. They are dynamic, open systems that change and evolve due to multiple interactions within and across the system, including positive and negative feedback, time delays and tipping points. CAS are self-organising and holistic.^1^ Health systems can be identified as CAS as they have many interconnected components (ie, agents, such as providers, patients, community, policy makers, and insurance agencies, and structures such as policies, norms, values, histories and capacities) that are constantly changing and adapting to changes.^2^

In the past 15 years, there have been increasing recommendations to use systems thinking (ST) in health systems because of their complex nature.^3 4^ ST is a discipline that can support us in making sense of CAS, it focuses on how components of a system are interconnected and how the system behaves.^5 6^

ST comprised theories, methods and tools that assist with addressing complex problems. It began in the 20th century and has been applied in countless disciplines, including biology, psychology, computer science and anthropology.^6^ It first emerged as a method for scientific investigation, but in the 1940s, it gained traction as a way to solve real-world problems related to World War II.^7^ Despite its long history, there is still no single agreed-upon definition of what ST is.^8^ Forrester and Richmond were among the first to define ST^7^. In Richmond’s article, we find the first complete definition of ST as ‘the art and science of making reliable inferences about behavior by developing an increasingly deep understanding of underlying structure’.^9^

Many definitions have followed and they all contain two common attributes: seeing the system holistically beyond just its components, and seeing the components in the context of the whole system.^7^ In other words, ST focuses on the holistic perspective of a system, and the observed behaviours that emerge from the interactions between the parts of the system.^7^

Using an ST approach has claimed to be beneficial for understanding and intervening in a health system.^3^ Trochim *et al*^10^ have suggested that ST can be used in health systems to create a more holistic view of financing, broaden non-traditional collaborations among disciplines, address the impact of social and political factors and identify barriers to implementing systems approaches.^10^ ST enables a change in mindset which allows individuals to solve complex problems through a holistic lens.^11^

Although there is a wealth of theoretical applications of ST in health systems, there is a gap between the conceptual use of ST and its actual application in the real world.^12–14^ Kwamie *et al*^15^ suggest that the application of ST needs to be documented better to build a stronger evidence base.^15^

One practical application of ST in health has been the Systems Thinking for District Health Systems (ST-DHS) initiative, which supported countries and health districts, to apply ST tools and practices to understand and intervene in their local health systems.^16^ As part of this initiative and to address the gaps in the application of ST in health systems, we have developed the ‘Systems Thinking for Health Actions (STHA)’ framework. This framework provides a structured approach to assessing the extent of application of ST in health actions, where ST terminology may not have been explicitly used. The framework aims to explore the application of ST principles and attributes in the formulation and evaluation of health actions. Furthermore, this new framework intends to be used in health actions as an operational checklist, with ST tools and methods that can be directly applied to the actions.

## Methods

The STHA framework was developed using a combination of a systematised literature review and expert inputs. The developed framework was then applied to a case study within the ST-DHS initiative to explore the ST characteristics in the COVID-19 response in Pakistan.

### Study setting

This research was conducted as part of a bigger project, the ST-DHS initiative, which was implemented in three countries: Botswana, Pakistan and Timor-Leste. The initiative provided two districts per country with ST tools and methods, aiming to improve local health systems with the new knowledge on how to apply ST.^16^

### Systematised literature review

A systematised literature review was conducted to create the STHA framework. This type of review contains elements of a systematic review but is missing elements that a systematic review would have, such as having two reviewers and registration of the review.^17^ A systematised review was chosen as time and resources were limited.

We developed a search strategy that included keywords to identify practical and theoretical uses of ST, as seen in online supplemental appendix 1. Using PubMed and Google Scholar, the first 50 results in each search term, based on the best match filter, were assessed for inclusion in the systematised review. The titles and abstracts were independently screened for relevance. If the abstract was pertinent, the full text was read and determined if it was to be included based on the content. Data were extracted and the ST tools and methods used in the manuscripts were compiled into a table (online supplemental appendix 2). They were then categorised into an Xmind map based on their intended uses as stated in the manuscripts.

10.1136/bmjgh-2022-010191.supp1 Supplementary data

10.1136/bmjgh-2022-010191.supp2 Supplementary data

The inclusion criteria were studies that explicitly mentioned and described ST concepts, tools or methods in theory or practice, were available in English and were accessible through the University of Basel. In addition, papers were included if they were published between 1 January 2009 and 31 December 2021. The year 2009 was chosen, as that is the year that ST in health systems gained traction with the publication of *Systems Thinking for Health Systems Strengthening*.^3^

### Framework development

The aim of the framework was to close the gap between ST theory and application.^12–14^ Stave and Hopper’s Taxonomy of Systems Thinking Objectives was used as the starting point of the framework. JT, DCM and CSF adapted the taxonomy based on the results of the systematised literature review and developed the first draft of the STHA framework.^8^ Once the ST characteristics were developed, we included definitions for each of them, as well as categorised the mapped ST tools by characteristic.

The first draft of the STHA framework was presented and discussed at two different virtual participatory workshops consisting of 14 health system researchers with extensive ST experience from Botswana, Pakistan, Switzerland and Timor-Leste. The first workshop consisted of individuals who had participated in the ST-DHS initiative, including the funder. The framework was presented and feedback collected regarding the categories, definitions and categorisation of the tools. The framework was then adapted and a second draft was presented in a workshop with health system researchers based at Swiss Tropical and Public Health Institute (Swiss TPH). The same approach was taken and the feedback was incorporated into a third draft of the framework.

JT, DCM and CSF made the final decision of what to include in the framework. The final draft was shared via email with all participants and those with interest were invited to participate in the write-up of this manuscript (see list of coauthors).

### Pakistan COVID-19 case study

Once we had the final STHA framework, we conducted a case study in Pakistan to validate and test if the framework adequately identified ST in Pakistan’s COVID-19 response. The case study was conducted with key informant interviews and a document review of the National Action Plan for Coronavirus Disease (COVID-19) Pakistan (National Action Plan). The key informant interviews were part of the rapid realist evaluation that was conducted for the ST-DHS initiative evaluation.^18 19^ The National Action Plan is a document that was developed by the Ministry of National Health Services, Regulation, and Coordination and Government of Pakistan to guarantee that the COVID-19 procedures for outbreak preparedness, containment and mitigation were followed.^20^ The Pakistan case study was chosen based on convenience as we were able to use the same data that were collected for the ST-DHS initiative.

#### Key informant interviews

The interviewees were two male and one female district health managers from the Islamabad district in Pakistan, representing 38% of total district health managers from the district.

The participants were purposively selected from the ST-DHS initiative and all participants provided consent to the interview. Saturation was discussed and due to the nature of the research, it was determined that three interviews were adequate to test the STHA framework.^21^ JT interviewed the health managers with whom she had no prior relationship. In addition to JT and the interviewee, a male researcher; MB, from Child Advocacy International, the local research partner, who had been involved in the implementation of the ST-DHS initiative, participated in all three interviews. The interviews lasted between 30 and 45 min and were conducted in English. The interviews were completed over Zoom, recorded with live transcription and stored on the Swiss TPH drive. The interviews were conducted using a semistructured interview guide that was created to evaluate the ST-DHS initiative. The guide was tested before implementation by conducting two practice interviews. Field notes were taken during and after the interviews. The participants did not review the interview quotation table before submitting the manuscript. No interviews were repeated.

### Patient and public involvement

This research did not contain any patient or public involvement.

## Results

### Identification of studies

**Figure 1:**
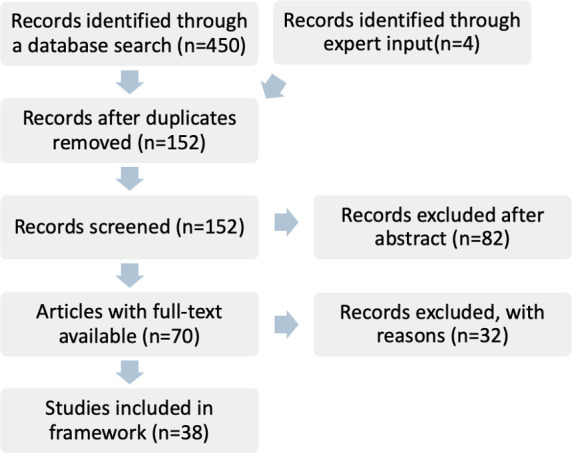
Systematised literature review results.

The initial search of the literature yielded 454 articles, which included four articles from expert input. After removing duplicates and screening the abstracts, 71 articles remained. Following a full-text review, 38 articles met the inclusion criteria and were included in developing the framework (figure 1). The 38 articles comprised systematic reviews (n=2), literature reviews (n=3), qualitative and/or quantitative studies (n=31) and commentaries (n=2). The research was conducted in a multitude of countries, including the USA (n=7), Australia (n=7), Ghana (n=1), Uganda (n=2), Zambia (n=1), Canada (n=1), India (n=1), Pakistan (n=1), Singapore (n=1) and Thailand (n=1). Additionally, there were nine studies with multiple countries and six did not have a specific country of research. The disciplines included in the review were health (n=36), food production (n=1), producing research (n=2), policymaking (n=1) and road traffic safety (n=1). The most commonly used ST tools were causal loop diagrams (n=27), systems dynamics modelling (n=12), agent-based modelling (n=8) and concept mapping (n=6) (table 1).

**Table 1:** Systems thinking tools

Systems thinking tool	n	Reference
Causal loop diagram	27	14 23 41 42 26 43 44 45 46 27 47 48 24 49 50 51 52 53 54 55 56 57 58 59 60 61
Systems dynamics modelling	12	8 11 23 41 26 27 48 54 62 63 64 65 66
Agent-based modelling	8	8 11 23 51 62 65 67 68
Concept mapping	6	8 11 41 52 57 65
Social network analysis	5	8 11 23 60 69
Group model building	4	52 58 60 70
Soft systems analysis	4	42 27 51 67
Behaviour over time graphs	3	57 63 70
Policy/document analysis	3	8 67 52
Process mapping	3	23 42 71
Scenario planning	3	11 23 27
Causal tree diagram	2	44 47
Conceptual frameworks	2	53 61
Media analysis	2	41 52
System map	2	60 61
Biomatrix tool	1	2
Iceberg tool	1	2
Innovation/change history	1	23
Logic models	1	52
Markov modelling	1	67
Multistakeholder dialogue	1	27
Participatory impact pathways analysis	1	23
Rich picture	1	72
Sociogram	1	41
Spatial patterning image	1	41
Systems archetypes	1	23
Viable systems model	1	41

### STHA framework

**Figure 2:**
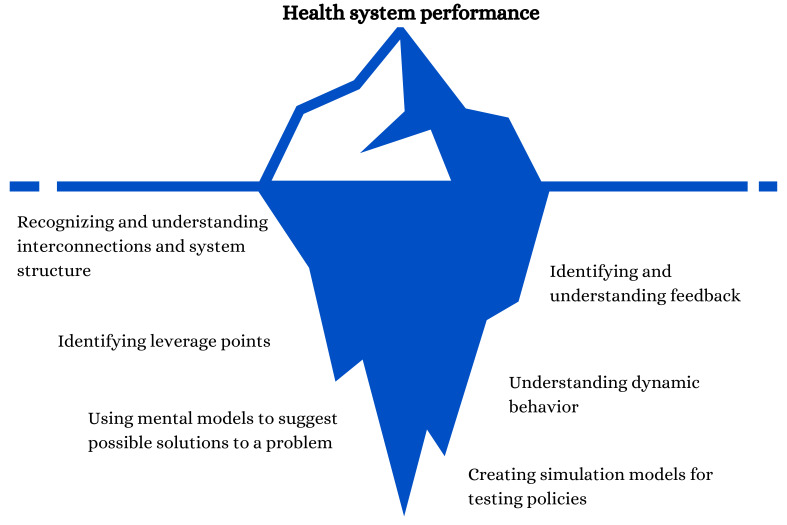
An iceberg model representing the varying complexity of the six systems thinking characteristics of the STHA framework.

An iceberg model, shown in figure 2, was chosen to represent this framework, as it allows a perspective shift from the visible health system performance and actions to how the application of ST can unveil underlying structures, patterns and behaviours of the system.^22^ The ST characteristics are not intended to be considered in order, rather the hierarchy of them only represents the varying levels of technical complexity required to apply them.^22^ The framework proposes a number of considerations that policy makers, managers, researchers or health practitioners can use to apply ST principles to the design, implementation and evaluation of health actions.

One of the first steps in moving from traditional linear thinking to ST is *recognising and understanding interconnections and system structure*. This characteristic comprised the recognising and understanding that health systems are composed of different interconnected parts. By recognising and understanding interconnections, we are able to gain insight of the key actors within a health system (including the agency of self within the system), as well as the overall system structure. It enables the stakeholders within the system to create shared goals and acknowledge how the health system can work together to implement successful health actions.^23^

The second ST characteristic is *identifying and understanding feedback in the system* since CAS (as health systems) are governed by feedback.^3^ Feedback is the cause and effect relationships that occur among the different elements in a system.^8^ It is critical to identify these relationships, both positive and negative, that occur among the parts of the health systems by recognising feedback loops and determining chains of causality within the system.^8^ This characteristic builds on recognising interconnections, as it recognises the connections and the directionality between them, and how indirectly an intervention can have a balancing effect on the desired outcome. In Uganda, a causal loop diagram demonstrated how feedback from government restrictions and policies influenced how the dual practice policy developed over time.^24^ Having an adequate feedback system in health actions allows the changing needs in the system to be identified and allows for interaction and seamless communication among all stakeholders, as well as preventing unintended outcomes.^24 25^

The third characteristic is *identifying leverage points*, which is a vital characteristic of ST, as these are areas where small changes can have a large impact.^26^ Identifying leverage points can help determine where to allocate scarce resources in most efficient ways or what small changes in health systems will yield substantial improvements in performance.^27^ In health systems, identifying leverage points systematically illuminates key areas to intervene, allowing for more targeted health actions.^28^ Glenn *et al*^29^ used qualitative data to develop a model of the neglected tropical disease system to identify potential leverage points for eliminating neglected tropical diseases.^29^

Approaching the more technically complex categories of the STHA framework is the characteristic *understanding dynamic behaviour*. As health systems are CAS, they are non-linear and dynamic over time.^29^ It is imperative to recognise the feedback loops^8^ and the interactions between the components of the health system that are responsible for generating patterns of behaviour that can change over time.^30^ Therefore, recognising dynamic behaviour can help determine the effect that behaviours from components of a health system have on the entire system.^8^

*Using models to suggest possible solutions to a problem* refers to the use of visualisations to display causality, feedback loops and variables to achieve the purpose of a health system. The visualised models do not depict a real-world system, rather how actors view the system. Developing systems models also include multistakeholder dialogue, which involves the process of defining the problem and delineating the boundaries of the system as well. By depicting the system, stakeholders can gain common understanding of the health system or health actions, as well as each other’s mental models, which facilitates finding potential solutions to the problem.^29^

Finally, *creating simulation models for testing policies* would arguably be the most complex analysis to integrate ST is *creating simulation models for testing policies*. Using ST simulation models helps translate multifaceted scientific findings into easy-to-understand outcomes.^31^ Simulation models should combine all previous characteristics and use qualitative and quantitative data to create a comprehensive model of the overall system.^8^ In health systems, simulation models can be used for assessing vulnerability, economic impact, measuring performance, emergency preparedness and how health systems are interdependent on other systems.^32^ Using simulation models is an important part of ST as it helps predict the impact a change will have and compare possible solutions to a problem.^8^

To transform the six ST characteristics into an operational framework for use in health actions, a checklist was created to explore the use of each characteristic in the formulation, design and evaluation of health actions.

The checklist, presented in table 2, shows each of the six framework characteristics in separate categories, as well as the corresponding checklist components and ST tools for each characteristic. The checklist was created as a guide to provoke ideas of how to apply an ST approach. The outlined characteristics and items do not have to be completely checked off to adequately incorporate the ST characteristic in the health action. Additionally, the ST characteristics are not intended to be considered in order.

**Table 2:** Systems Thinking for Health Actions checklist

Systems Thinking for Health Actions checklist	Relevant systems thinking tools
**Recognising and understanding interconnections and system structure.**	Stakeholder mapping/analysis. Social network analysis. Analysis of industry documents, tactics and strategies. Stakeholder interviews. Sociogram. Process mapping. Causal loop diagram. Logic models. Reflective practice.
Identified components of the health system.	
Visually or textually showed the connections between components of the health system.	
Conducted focus groups and/or interviews of key stakeholders to understand the health system better.	
Invited other relevant sectors to participate in the design of the intervention.	
Recognised the need for stakeholder involvement.	
**Identifying and understanding feedback.**	Causal loop diagramming. Markov modelling. Stakeholder interviews. Agent-based modelling. Stock and flow diagrams. Systemic policy analysis. Logic models. Sociogram.
Visually or textually addressed the feedback loops that exist in the health system.	
Identified the positive and negative effects one component of the health system has on other components.	
**Identifying leverage points.**	Iceberg tool. Scenario planning. Decision tree modelling. Logic models. Group model building. Systems dynamics modelling. Focus groups and stakeholder interviews. Business process mapping/discrete event modelling.
Determined the root causes of a problem through pictorial or written mapping.	
Attempted to identify gaps.	
Determined the key actions for leverage points.	
**Understanding dynamic behaviour.**	Causal loop diagram. Behaviour over time graphs. Dynamic thinking. Innovation/change management history. Systems archetypes. Stock and flow diagram. Causal loop diagram with variable distinction. Table differentiating the variables.
Showed how a problem changes over time.	
Addressed problems between components of the health system.	
Predicted the impact a change to one component of the health system has on the rest of the system.	
Identified how components of the health system change over time.	
Addressed path dependence.	
Developed a mechanism to identify emerging behaviours in the health system.	
**Using models to suggest possible solutions to a problem.**	Conceptual model. Theory of change.
Explained the expected outcome of and action on the health system.	
Explained why the expected outcome is anticipated.	
Used a diagram, descriptive text or a pictorial model to represent the system.	
**Creating simulation models for testing policies.**	Agent-based models. Systems dynamics models. Scenario planning models. Simulation models.
Used qualitative and quantitative data to create models.	
Used identified leverage points to test a change.	
Interpreted model outcomes.	
Compared solutions from different leverage points.	

### Application of the STHA framework to the COVID-19 response in Pakistan: a case study

#### Data analysis

The National Action Plan and key informant interviews from the COVID-19 response in Pakistan were analysed using thematic analysis with a deductive approach.^33^ We applied the STHA framework to analyse the data. First, the interview transcripts and National Action Plan were read and a codebook was created using the STHA framework as a guide. The text was highlighted using the corresponding themes from the codebook. With the relevant areas of the transcript selected, the highlighted text was applied to the six attributes of the framework to determine if and where the selected text fit best. Microsoft Excel was used to manage the data extracted from the transcripts and the data were coded by JT.

The Consolidated Criteria for Reporting Qualitative Research checklist was consulted for reporting this qualitative research.^34^ The participants gave verbal consent to participate in the interviews, which were recorded.

The National Action Plan *identified the system structure* by listing the key stakeholders and sectors (pp 18–20) involved in the COVID-19 response (including those outside the health system) and the actions expected to be taken by each one of them (p 104). Furthermore, the National Action Plan also textually showed the *connections and emergent behaviour between the components of the system* through ‘rapidly establishing and strengthening coordination to deliver strategic, technical and operational support through existing mechanisms and country partnerships’ (p 11) and creating a ‘policy framework for federal, provincial and regional stakeholders for building capacity to prevent, detect and respond to any events due to COVID-2019 or other novel pathogens with pandemic potential in Pakistan’ (p 9). The action plan also recognised the need for stakeholder involvement and included the relevant stakeholders in the COVID-19 surveillance system (p 14) and the development of the Risk Communication and Community Engagement initiative (p 17).

Respondents 2 and 3 identified the use of process mapping in the district, which assisted with identifying the stakeholders involved in the system. Respondent 3 mentioned the value of mapping stakeholder connections and roles to see how they impact each other. Additionally, respondents 2 and 3 also described identifying *emergent behaviour* in the system using reflective practice.

*To identify and understand feedback* the COVID-19 action plan implemented a monitoring and evaluation plan for constant improvement of the COVID-19 response (p 20). This included a parallel evaluation to continually point out areas for improvement. Additionally, the action plan identified the effect that one component of the system (specifically funding) can have on other components by acknowledging the roles that funding has on surveillance structures, data, laboratory diagnostic capacity, case management, stockpiling and logistics, infection prevention and control, burial policy and risk communication (p 17).

Respondent 3 explained how *understanding the connections* between the components of the process map assisted in realising the effect one component of the system has on another: ‘[process mapping] *gave a very clear pattern of how things were how many stakeholders were involved in everything, and the reflective processes, has had a lot as well you know, to be honest.*’

The COVID-19 action plan in Pakistan *identified the gaps and leverage points* in their COVID-19 system response. ‘Assessments of risks and capacities to determine priorities for emergency preparedness’ were conducted (p 12). Among the gaps mentioned in the action plan were the capacities of case management, risk communication and infection prevention and control at health facilities (p 13), and the disease outbreak management system needing to be strengthened (p 14). The action plan also identified 19 key action areas or leverage points where actions could help minimise the spread of COVID-19 (pp 20–31).

The action plan also *addressed the system’s dynamic behaviour* by predicting how preparedness in the initial phase and strict containment in the second phase would determine the impact of the virus (p 8). It also mentioned ‘strengthening and reforms of the organizational, structural and coordination mechanisms to ensure the maximum level of preparedness over time’ (p 10).

In summary, four out of the six characteristics of the STHA framework were identified in Pakistan’s COVID-19 response.

## Discussion

ST has been a commonly used approach in various disciplines to address multifaceted problems in CAS.^6^ Adopting an ST approach is an attractive method in the field of health systems, but there is still a lack of understanding of the practical uses of ST.^35^ This framework aimed to bridge the gap between theoretical ST and practical ST.^13 33^ Six key ST characteristics were identified in the framework: (1) recognising and understanding interconnections and system structure, (2) identifying and understanding feedback, (3) identifying leverage points, (4) understanding dynamic behaviour, (5) using mental models to suggest possible solutions to a problem and (6) creating simulation models to test policies. We identified two potential applications for the framework and checklist: (1) Prospectively, to support in the design or implementation of health actions. Applying the framework prospectively can be done as a guide to translate ST concepts into practical steps that can be integrated in the design or implementation of a health action. (2) Retrospectively, to investigate where ST was applied and where it can be further applied the next time. The case study provided in this article is a retrospective example of applying the STHA framework.

We developed the STHA checklist to act as a guide to explore the application of ST prospectively or retrospectively in health actions. Checklists ease work in demanding or tense situations and have been increasingly used in healthcare.^36^ Checklists help promote active cooperation and communication among stakeholders.^37^ Therefore, using our checklist to assist in translating the application of ST concepts into practical steps can be beneficial. The checklist, which should not be taken as a strict and linear document, can help relieve some of the barriers to applying ST, such as many stakeholders understanding that ST requires sophisticated and resource-intensive interventions, as well as the lack of knowledge on how to start using ST.

The application of the STHA framework to the COVID-19 response in Pakistan revealed that the less complex characteristics were applied throughout the response despite the exact ST terminology not necessarily being mentioned. In our interviews, we identified several ST tools being used by district health officials, such as reflective practice or process mapping.^38^ The district officials and the research team used these tools as part of the ST-DHS initiative. In addition to the use of ST tools, we also identified other ST characteristics in the COVID-19 response (such as the understanding of dynamic behaviour in the National Action Plan) that were not influenced by the ST-DHS initiative. This highlights how ST is often used without being explicitly mentioned. Given the high complexity and changing environment of the pandemic, the health officials had to apply holistic responses.

The COVID-19 pandemic presented a crisis where policy makers had to quickly respond to a threat without knowing the exact extent of it.^35^ Therefore, the lack of application of the other three categories in the framework could be due to the time-sensitive nature of the pandemic and the lack of time between creating policies and implementing them. In previous literature, several barriers have been described to the application of ST approaches such as assumed costliness, lack of understanding, competing political interests across health and non-health actors, which often lead to prioritisation of vertical programmes, work in silos and difficulty ensuring meaningful multistakeholder involvement.^39^ Although the framework cannot mend all of these perceived barriers, it can assist in informing health action creators with more knowledge of using ST tools. Additionally, by revealing that ST exists in many health actions, it can show that the use of ST does not have to be costly, as it is already being applied to health actions without further costs.

In the Pakistan case study, we were able to validate the framework and determine that the framework was effective in identifying ST characteristics in the action plan and key informant interviews. However, sometimes it was difficult to determine if a section of text adequately included an ST characteristic. To lessen these uncertainties, further defining the characteristics will ease the use of the checklist in the future. Additionally, the checklist was only applied retrospectively to assure its use in assisting with the application of ST in impending health actions, the framework should also be validated in prospective cases.

To create the framework, the literature review was not limited to ST in health systems, rather other disciplines were also included (ie, road traffic safety, policymaking and food production). The limitations of this method were that not all disciplines were included in the search, rather disciplines that were previously well known for ST and expert inputs. In addition, the search was limited to English language, manuscripts that could be accessed through the University of Basel, manuscripts that were found on PubMed and Google Scholar and there was only one reviewer. Searching only two academic research databases may have limited the number of results retrieved for the literature review, thereby missing manuscripts that should have been included.^40^ Therefore, when developing the STHA framework further, a more extensive literature review could be conducted, with the number of disciplines and databases expanded. In addition, expert inputs from other systems thinkers would be helpful to expand the checklist developed for each characteristic of ST. The interviews conducted for testing the framework were specific to the ST-DHS initiative, meaning that the respondents already had knowledge of ST, which could have been a bias in their answers. The framework was also applied to only three interviews, with the respondents having similar positions in the district health system, which may have limited the ideas that interviewing other positions may have added. Expanding the application of the STHA framework to more interviews and with a wider range of positions could assist in further developing it. This was a first pilot of the STHA framework. In order for it to be relevant for a wider set of topics, actions and stakeholders, as well as context, further research is needed to identify how to improve it.

## Conclusion

This paper aimed to bridge the gap between theoretical and practical ST in health actions. Stave and Hopper^8^ created an excellent starting point for bridging this gap by identifying the level of ST in individuals in their Taxonomy of Systems Thinking Objectives. The STHA framework has made additional progress in closing this gap by creating a tangible checklist for designing, implementing and evaluating health actions. The STHA framework is a new, innovative way to apply ST. This framework can be used retrospectively, and it can be a guide in the development of health actions to explore where ST can still be applied. Further research is needed to ensure the STHA framework reaches its full potential.

## Data Availability

No data are available.
