# Supplementary material for: Development of the Systems Thinking for Health Actions framework: a literature review and a case study
Source: BMJ Glob Health. 2023 Mar 17;8(3):e010191. doi: 10.1136/bmjgh-2022-010191 (PMC10030275; doi:10.1136/bmjgh-2022-010191)
Supplement: Supplementary data [file bmjgh-2022-010191supp001.pdf]

Search Strategy

Electronic databases:

1. PubMed
2. Google Scholar

Search dates: Search in all databases were conducted in December 2021

Restrictions on publication period: The results shown here were restricted to after January 2009.

Restrictions on language: Articles published in English

Types of studies: No restriction

Exclusion criteria: languages other than English and publication date earlier than January 2009

Inclusion criteria: Systems thinking tools and methods were used in theory or in practice

The first 50 results in each search were screened. Additionally, 4 papers were added from expert input.

Search Strategy
Database: PubMed

----

1. **“Systems Thinking” Health Interventions OR “Systems Thinking” Health Initiatives OR “Systems Thinking” Application in Health OR Health “Systems Thinking” Analysis OR Health “Systems Thinking” Approach** Search results: 452
2. **“Systems Analysis” Health Interventions OR “Systems Analysis” Health Initiatives OR “Systems Analysis” Health Application** Search results: 211
3. **“Systems Thinking” Lens OR “Systems Thinking” Perspective** Search results: 197
4. **“Systems Thinking” Tools OR “Systems Thinking” Methods OR “Systems Thinking” Skills**
5. **OR “Systems Thinking” Policies** Search Results: 402
6. **“Systems Thinking” Climate Change OR “Systems Thinking” Traffic Safety OR “Systems Thinking” Public Health** Search Results: 625
7. **(“Systems Thinking” OR “Systems Science” OR “Complexity Science” OR “Complex Systems” OR “System Dynamics” OR “Systems Modelling”) AND “Health”** Search Results: 4,667

Database: Google Scholar

1. **((“Systems Thinking” Interventions OR “Systems Thinking” Initiatives OR “Systems Thinking” Application OR “Systems Thinking” Analysis OR “Systems Thinking” Approach) AND “Health”) Search Results: 17,000**
2. **((“Systems Thinking” Lens OR “Systems Thinking” Perspective) AND “Health”) Search Results: 16,900**
3. **((“Systems Thinking” Tools OR “Systems Thinking” Methods OR “Systems Thinking” Skills OR “Systems Thinking” Policies) AND “Health”) Search Results: 17,500**
