# Supplementary material for: Development of the Systems Thinking for Health Actions framework: a literature review and a case study
Source: BMJ Glob Health. 2023 Mar 17;8(3):e010191. doi: 10.1136/bmjgh-2022-010191 (PMC10030275; doi:10.1136/bmjgh-2022-010191)
Supplement: Supplementary data [file bmjgh-2022-010191supp002.pdf]

| Title | Country of research | ST methods used | Study | Results | Key elements | Domain(s) involved |
|---|---|---|---|---|---|---|
| **Systematic Reviews** | | | | | | |
| The application of systems thinking concepts, methods, and tools to global health practices: An analysis of case studies doi:10.1111/jep.12842 (Wilkinson et al., 2018) | United States, United Kingdom, Ghana, Canada, China, Australia, Uganda, Bosnia and Herzegovina | Agent based modelling, social network analysis, scenario planning, systems dynamics modeling (stock and flow diagrams, CLD), innovation/change history, participatory impact pathways analysis, process mapping, stock and flow diagrams, systems archetypes | Review | Common ST methods and tools were largely underutilized. Four themes were identified: the importance of interconnectedness of a system, attributes of leaders in CAS, and the benefits and barriers of using ST | Systematic review of 36 case studies that used ST | Global health |
| Thinking about complexity in health: A systematic review of the key systems thinking and complexity ideas in health doi: 10.1111/jep.12856 (Rusoja et al., 2018) | Various | Systems dynamics modeling, CLD, agent-based modeling, social network analysis, concept mapping, scenario technique | Review | ST remains largely a theoretical approach and there is a need for further practical application of it. | A systematic review that aimed to identify key terms, concepts, and methods in ST literature. | Healthcare |
| **Reviews** | | | | | | |
| Systems science and oral health: Implications for Dental Public Health? doi:10.1922/CDH_4470Broomhead08 (Broomhead, 2019) | Not specified | System dynamics simulations, agent-based models, network analysis, soft-systems analysis, Markov modelling, policy relevant analysis | Review | ST offers another dimension to the dental field and can be beneficial in addressing complexity and advancing knowledge of key mechanisms and evolving properties of understanding and promoting dental health. | An overview of how systems science can be used to address complexity problems in the field of dental health and the benefits that ST approaches can have | Dental Health |

| | | | | | | |
|---|---|---|---|---|---|---|
| Applications of systems modelling in obesity research<br>doi: 10.1111/obr.12695<br>(Xue et al., 2018) | Not specified | Agent based modelling and systems dynamics modelling | Review | Found that there is still a gap between theory and application of systems approaches in public health. | Studied the uses of systems modelling and agent-based modelling in obesity research. | Obesity research |
| Evaluation of public health interventions from a complex systems perspective: A research methods review<br>doi:<br>10.1016/j.socscimed.2021.113697<br>(McGill et al., 2021) | Not specified | CLD, concept mapping, stock and flow diagrams, viable systmes model, sociogram, multi-media models, network diagram and spatial patterning image | Review | Found a wide range of methods used in complex systems and categorized them into mapping, modelling, network analysis and system framing. | Conducted a review of public health interventions to classify and describe methods used in complex public health systems. | Public health |
| **Individual studies** | | | | | | |
| A systems approach to assessing complexity in health interventions: an effectiveness decay model for integrated community case management<br>doi:10.1080/16549716.2020.1794106<br>(Karim et al., 2020) | Low and middle income countries | Decision tree modelling, process mapping, CLD, soft systems methodology | Intervention | The approach provided insights on many aspects of the program it studied and how its elements interacted with each other, and how it fit within the overall health system | A robust systems approach that proposes the use if system thinking frameworks, concepts and tools | Child health |
| Advancing the application of systems thinking in health: understanding the dynamics of neonatal mortality in Uganda<br>doi:10.1186/1478-4505-12-36<br>(Rwashana et al., 2014) | Uganda | CLD and systems dynamics modeling | Analysis | The CLDs revealed feedback loops and high leverage points to be seen | Devleoped CLDs to display the demand and supply side issues in health services | Neonatal mortality and maternal health |

| | | | | | | |
|---|---|---|---|---|---|---|
| Understanding a successful obesity prevention initiative in children under 5 from a systems perspective doi:10.1371/journal.pone.0195141 (Owen et al., 2018) | Australia | CLD | Analysis | The CLD allowed for the complexity of a community based intervention to be represented and understood from feedback loops | Developed a CLD to represent stakeholder perceptions of an intervention | Childhood obesity prevention |
| Advancing the application of systems thinking in health: realist evaluation of the Leadership Development Programme for district manager decision-making in Ghana doi:10.1186/1478-4505-12-29 (Prashanth et al., 2014) | Ghana | CLD and causal tree diagram | Evaluation | The CLD showed that the program did not deviate much from the predicted implementation of the program. | An evaluation of a leadership program, using a CLD to explain the interactions between contexts, outcomes and mechanisms of the program | Leasdership development in Healthcare |
| Advancing the application of systems thinking in health: advice seeking behavior among primary health care physicians in Pakistan doi: 10.1186/1478-4505-12-43 (Malik et al., 2014) | Pakistan | Social network analysis | Analysis | The use of the social network analysis showed that doctors did not contact the correct representative when facing a difficult case and the key stakeholder interviews revealed gaps and problems in the system from different stakeholder point of views. | Cross-sectional study that analysed the existing primary health care system and explore how it could be strengthened | Primary Healthcare |
| Advancing the application of systems thinking in health: understanding the growing complexity governing immunization services in Kerala, India doi: 10.1186/1478-4505-12-47 (Varghese et al., 2014) | India | CLD | Analysis | The CLD assisted in the identification and interpretation of the feedback loops that emerged in the analysis. It also helped identify unintended consequences | Explored underlying factors in vaccination coverage and carried out content analysis of the data by looking at | Immunization services |

| | | | | | | |
|---|---|---|---|---|---|---|
| | | | | and unexpected phenomena. | features of the complex adaptive systems. With this data, a CLD was created to show the interactions among the key actors and elements of the system. | |
| Building a systems thinking prevention workforce doi:10.1002/hpja.325 (Bensberg et al., 2020) | Australia | CLD | Analysis | The CLD revleaded how cause-effect relationships were drawn from the findings and how they contributed to systems thinking capacty building. | Conducted semi-structured interviews on particpants understanding of the ST, the data from the interviews were used to create a CLD. | Workforce systems thinking development in healthcare |
| Using a 'rich picture' to facilitate systems thinking in research coproduction doi:10.1186/s12961-019-0514-2 (Conte & Davidson, 2020) | Australia | Rich pictures | Analysis | The rich picture allowed for the whole system to be seen and for key stakeholders to take part in the inquiry process and to express data in a way that might not be possible through speech and writing. | Used systems thinking principals to examine the dynamics between an IT system and the intevention it was designed to monitor. | Research coproduction |
| Systems thinking for health emergencies: use of process mapping during outbreak response doi:10.1136/bmjgh-2020-003901 | Sierra Leone, the Democratic Republic of | Process mapping | Intervention | In all three case studies, the time for reporting the specific disease to the intended audience was | Used a four-step process mapping methodology for disease outbreaks | Disease outbreaks |

| | | | | | | |
|---|---|---|---|---|---|---|
| (Durski et al., 2020) | Congo (DRC) and Nigeria | | | drastically lowered after the process mapping exercise was implemented and the time for reporting data was also decreased. | in three different case studies | |
| Systems approaches to population health in Canada: how have they been applied, and what are the insights and future implications for practice? doi:10.17269/s41997-019-00230-3 (Zukowski et al., 2019) | Canada | Soft systems methodology, CLD, scenario planning, multi-stakeholder dialogue, systems dynamics modelling | Analysis | Qualitative methods are effective involving diverse perspectives to develop a complete hypothesis of a system and quantitative models can help determine where the best place in the system is to intervene. | A rapid review of literature to identify case studies of systems thinking in Canada. | Population health |
| Using systems thinking in state health policymaking: an educational initiative doi: 10.1057/hs.2013.17 (Minyard et al., 2014) | USA | Behavior over time graphs, stock and flow maps, systems dynamics models | Intervention | 93 legislators participated in the course, where the participants were receptive to the systems thinking tools. | A systems thinking certificate program to improve policymaking processes in Georgias legislators. | Policymaking |
| Advancing the application of systems thinking in health: provider payment and service supply behaviour and incentives in the Ghana National Health Insurance Scheme – a systems approach doi: 10.1186/1478-4505-12-35 (Agyepong et al., 2014) | Ghana | CLD and causal tree diagrams | Analysis | There is a need for holistic designs and implementations, therefore a systems approach is needed rather than a linear approach. | Used mixed methods to evaluate the payment methods in the Ghana health system | Health system |

| | | | | | | |
|---|---|---|---|---|---|---|
| A Systems Thinking approach for responding to the COVID-19 pandemic<br>doi: 10.26719/emhj.20.090<br>(Hassan et al., 2020) | South Korea, New Zeeland and Jordon | Iceberg tool and biomatrix tool | Commentary | The systems thinking approach is supported through a review of literature and the COVID-19 measures taken in the countries observed. | Applied the Iceberg Tool and Systems Thinking Biomatrix Tool to identify leverage points in the COVID-19 response | COVID-19 |
| Systems thinking perspectives applied to healthcare transition for youth with disabilities: a paradigm shift for practice, policy and research<br>doi: 10.1111/j.1365-2214.2011.01313.x<br>(Hamdani et al., 2011) | Various | Systems dynamics model, CLD and stock and flow diagram | Intervention | The use of systems thinking can assist in determining areas leverage points for interventions and the intended and unintended impacts the interventions will have on the system. | Introduced several systems thinking concepts to healthcare transition for youth with disabilties | Health system |
| Advancing the application of systems thinking in health: exploring dual practice and its management in Kampala, Uganda<br>doi: 10.1186/1478-4505-12-41<br>(Paina et al., 2014) | Uganda | CLD | Analysis | The CLD illustrated that dual practice occurs due to incentives within the public and private sector. | Conducted a document review, qualitaive interveiws and a qualitative survey to explore the use of dual practice in Uganda. From the data collected, a CLD was created to show the feedback loops in dual practice. | Health care providers |
| Emergency department crowding in Singapore: Insights from a systems thinking approach<br>doi: 10.1177/2050312116671953 | Singapore | CLD | Analysis | The CLD was a great tool to observe the structure of emergency department crowding in Singapore. | Created a CLD after consuting with emergency department | Emergency care |

| | | | | | | |
|---|---|---|---|---|---|---|
| (Schoenenberger et al., 2016) | | | | | experts, a literature review and direct observation. | |
| Understanding the dynamics of obesity prevention policy decision-making using a systems perspective: A case study of Healthy Together Victoria<br>doi: 10.1371/journal.pone.0245535<br>(Clarke et al., 2021) | Austrailia | CLD | Analysis | The CLD revealed recurrent associations and feedback mechanisms between the components of the policy systems. | Created a CLD from interviews and a document analysis from the Healthy Together Victoria Initiative | Obesity prevention policies |
| Using systems science to understand the determinants of inequities in healthy eating<br>doi: 10.1371/journal.pone.0188872<br>(Friel et al., 2017) | Austrailia | CLD, agent-based models, network analysis and soft systems methodology | Analysis | The final CLD made it possible to organize and analyze comply information with a holistic system perspective. | Used a group of policy experts to build a systems model and mind maps to created a final CLD. | Healthy eating |
| Rethinking health systems strengthening: key systems thinking tools and strategies for transformational change<br>doi: 10.1093/heapol/czs090<br>(Swanson et al., 2012) | Not specified | Concept mapping, social network analysis, agent-based modelling, system dynamics modelling, systemic policy analysis | Commentary | Suggests three overarching themes of using ST tools: collaboration across disciplines, sectors and organizations; ongoing, iterative learning; and transformational leadership | Agrues that key ST tools and strategies have the possibility to transform health systems. | Health systems |
| Systems Thinking as a Framework for Analyzing Commercial Determinants of Health<br>doi: 10.1111/1468-0009.12339<br>(Knai et al., 2018) | Not specified | CLD, conceptual modeling, stakeholder analysis, document analysis, media analysis, group model building, logic models | Analysis | Found that applying a systems perspective was beneficial and can be used in other complex public health issues. | Examined how a systems perspective can be useful to analyzing the commercial | Determinants of health |

| | | | | | | |
|---|---|---|---|---|---|---|
| | | | | | determinents of noncommunicable diseases. | |
| Application of systems thinking: 12-month postintervention evaluation of a complex health system intervention in Zambia: the case of the BHOMA doi: 10.1111/jep.12354 (Mutale et al., 2017) | Zambia | Systems-thinking guided analysis framework and CLD | Intervention | The ST approach used assisted in evaluating the complex intervention. | Conducted a qualitative study and used a systems thinking conceptual framework to analyze the intentional and unintentional consequences of the intervention. | Health system |
| Using Behavior Over Time Graphs to Spur Systems Thinking Among Public Health Practitioners doi:10.5888/pcd15.170254 (Calancie et al., 2018) | USA | Behavior over time graphs and group model building | Intervention | Most practitioners involved in the learning of behavior over time graphs found it a useful tool for engaging stakeholders. | Suggests the use of beheavior over time graphs to describe a variety of public health issues to public health practicioners | Public health |
| Understanding the Problem of Access to Public Health Insurance Schemes among Cross-Border Migrants in Thailand through Systems Thinking doi:10.3390/ijerph17145113 (Kunpeuk et al., 2020) | Thailand | Dynamic simulation model, CLD, stock and flow diagram | Application /Intervention? | The CLD and stock and flow diagram helped identify the dynamics of migrants accessing health insurance in Thailand. | Applied ST to explore the factors limiting public health insurance access to migrants in Thailand. | Health insurance |
| Moving towards culturally competent health systems for migrants? Applying systems thinking in a | Malaysia | CLD | Analysis | The CLDs helped identify four major themes affecting micro-level interactions in the health system. | Conducted key stakeholder interviews and a | Health system |

| | | | | | | |
|---|---|---|---|---|---|---|
| qualitative study in Malaysia and Thailand doi: 10.1371/journal.pone.0231154 (Pocock et al., 2020) | | | | | document review to create CLDs . | |
| Applications of Systems Science to Understand and Manage Multiple Influences within Children's Environmental Health in Least Developed Countries: A Causal Loop Diagram Approach doi: 10.3390/ijerph18063010 (Brereton & Jagals, 2021) | LDC | CLD | Analysis | The CLD helped identify important feedback loops between different variables that effect children's environmental health. | A literature review was conducted to determine variables for a CLD. | Children's environmental health |
| Capturing complexity in work disability research: application of system dynamics modeling methodology doi: 10.3109/09638288.2015.1031291 (Jetha et al., 2016) | USA | Systems dynamics modeling | Application | The system dynamics model captured the complexity of work disability and shows a visual of the dynamic relationships that effect work disability. | Applied a systems thinking lens to work disability and created a system dynamics model. | Work disability research |
| Systems Thinking Tools as Applied to Community-Based Participatory Research: A Case Study doi: 10.1177/1090198111430708 (BeLue et al., 2012) | USA | CLD, behavior over time graphs, and concept mapping | Intervention Planning | The use of the tools may help understand the dynamics of a community and may help with sustainability and effectiveness of interventions. | Used case studies to demonstrate the use of systems thinking tools. | Community-based participatory research |
| Systems thinking and complexity: considerations for health promoting schools doi: 10.1093/heapro/dav109 (Rosas, 2017) | USA | Network analysis, group model building, and CLD | Analysis | Using ST can assist in addressing the challenges in promoting health in schools. | Used ST to determine challenges in promoting health in schools through the exploration of four system | School health |

| | | | | | | |
|---|---|---|---|---|---|---|
| | | | | | thinking areas: knowledge, networks, models and organizing. | |
| Translating systems thinking into practice for community action on childhood obesity<br>doi:10.1111/obr.12865<br>(Allender et al., 2019) | Australia | CLD | Intervention | The creation of CLDs allowed the community to visualize the community's systems and see where they feel action is possible. | Used a system lens to describe a obesity intevention, specifically the Fister-Fishman's systems frameowork. | Childhood obesity |
| A Systems Thinking Methodology for Studying Prevention Efforts in Communities<br>doi:10.1007/s11213-020-09544-7<br>(Riley et al., 2021) | Australia | Social network analysis, system map, community workshop, group model building, CLD and system inventory | Intervention | Allowed for a wide range of ST methods to be applied, while allowing adaption for the specific contexts. | Created a methodolgy that combined ST tools and systematic inquiry processes. It included four domains of inquiry. ST tools were applied to investiagte each domain of inquiry. | Public health |
| Complex systems thinking and current impasses in health disparities research<br>doi: 10.2105/AJPH.2011.300149<br>(Diez Roux, 2011) | USA | Concept mapping, agent-based models, and system dynamics models | | ST can help health disparities research move beyond current impasses that are in place. | Provided illustrative examples of how ST tools can help to address unanswered questions in various examples. | Health disparities research |
| Systems Thinking in the Context of Road Safety: Can Systems Tools Help | USA | System map, CLD and 5 r's framework | | ST tools can help to implement the Safe Systems | Suggests a Safe Systems approach | Road traffic safety |

| | | | | | | |
|---|---|---|---|---|---|---|
| us Realize a True "Safe Systems" Approach?<br>(Naumann et al., 2020) | | | | concepts holistically. ST tools help produce a unified and cohesive view of the problem  the multi-component factors and interactions of the underlying problem and the larger system we are working within. | for road safety practice using ST tools. | |
| Agent-Based Modeling in Public Health: Current Applications and Future Directions<br>doi: 10.1146/annurev-publhealth-040617-014317<br>(Tracy et al., 2018) | Not specified | Agent-based modelling | Analysis | Using agent-based models in public health can improve model validation, and better inform research practice and policy. | Explored how agent-based models can improve public health | Public Health |
